# “There Is So Much More for Us to Lose If We Were to Kill Ourselves”: Understanding Paradoxically Low Rates of Self-Harm in a Socioeconomically Disadvantaged Community in London

**DOI:** 10.1177/1049732320957628

**Published:** 2020-09-15

**Authors:** Catherine Polling, Charlotte Woodhead, Hannah Harwood, Matthew Hotopf, Stephani L. Hatch

**Affiliations:** 1 Institute of Psychiatry, Psychology and Neuroscience, King’s College London, London, United Kingdom; 2 South London and Maudsley NHS Foundation Trust, London, United Kingdom

**Keywords:** London, United Kingdom, mental health, self-harm, stress process, deprivation, place, ethnicity, qualitative, interviews, focus groups, thematic analysis

## Abstract

London has unexpectedly low overall rates of self-harm in public health data and contains highly deprived areas with these paradoxically low rates. Qualitative data were collected via interviews and focus groups with 26 individuals living and working in one such area. Using the Stress Process Model, we explore why this ethnically diverse community, which is exposed to multiple, chronic stressors, might nonetheless appear to have low rates of self-harm. Participants described significant impacts of stressors on the mental health of people locally. These were partly buffered by social resources related to community solidarity and a culture of self-reliance. However, identifying oneself as mentally ill through being known to have self-harmed was seen as highly risky, diminishing a person’s social status and exposing them to additional stressors from the community and services. Consequently, people tended to hide distress, respond with behaviors less linked to mental illness, and avoid mental health services.

## Introduction

Self-harm is a behavior that occurs in response to distress, often but not necessarily as a symptom of a range of mental disorders (Haw et al., 2001). In this study we use the United Kingdom’s National Institute for Health and Care Excellence (NICE) definition of self-harm: “any act of self-poisoning or self-injury carried out by an individual regardless of motivation [in terms of suicidal intent]” (NICE & National Collaborating Centre for Mental Health, 2004).^1^ Rates of self-harm, both non-fatal and suicide, are known to vary substantially between geographical areas and populations of different social statuses (Cairns et al., 2017; Platt, 2016) and are consistently associated with individual- and community-level socioeconomic deprivation (Cairns et al., 2017; Congdon, 2013) indicating an important role for social context. However, the relationship between self-harm and deprivation is not as uniform as these epidemiological findings suggest. Within the United Kingdom, London has above national levels of socioeconomic deprivation and so would be expected to have high rates of self-harm. However, routine statistics available for self-harm admissions to general hospitals do not support this (Congdon, 2013); rates in the capital are half the national rate for England (Public Health England, 2020). Quantitative studies using routine clinical data from 2009 to 2016 for South London to examine geographical patterning of small-area rates of self-harm within the city have shown that patterns were not explained by area-level socioeconomic deprivation and that some deprived inner-city areas had these paradoxically low rates (Polling et al., 2019a; Polling et al., 2019b).

In this study, we focus on a specific, local area within London where rates of self-harm are low compared with the surrounding city despite very high levels of socioeconomic deprivation. We aim to understand the relationship between socioeconomic deprivation and self-harm in the study area, by considering how experiences within the community shape the way stressors are understood and experienced, and the resources available to cope with them. We use interviews and focus groups with people with in-depth local knowledge to focus on experiences of stress, distress, and self-harm within the community.

### Stress Process Model

**Figure 1.**
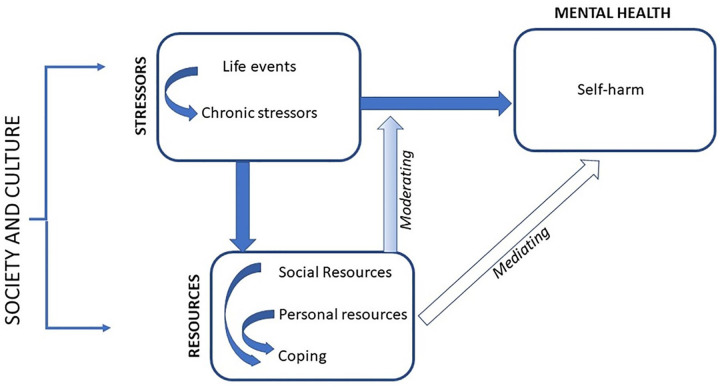
The Stress Process Model. *Source*. Adapted from Pearlin et al. (1981) and Aneshensel and Avison (2015).

We used Pearlin’s Stress Process Model (Pearlin, 2010; Pearlin et al., 1981), illustrated in Figure 1, to conceptualize how social context at both the macro and community level may influence an individual’s risk of self-harm, and why exposure to apparently similar socioeconomic stressors may lead to different mental health outcomes in different contexts. The Stress Process Model defines stressors as external threats or challenges which exceed individuals’ ability to respond or prevent them attaining sought after ends (Aneshensel & Mitchell, 2014; Pearlin, 2010). These include acute life events, which result in disruptive or undesirable change, and chronic stressors where difficult situations persist over time (Aneshensel & Avison, 2015). Stress is the response, both physiological and psychological, evoked in individuals by these stressors, which amounts to distress when it causes mental health symptoms such as anxiety, results in harmful behavior, or otherwise disrupts individuals’ ability to function (Wheaton et al., 2013).

The conditions that characterize socioeconomically disadvantaged areas, including insecure employment, poor quality, and over-crowded housing and high levels of crime (Turney et al., 2013), expose individuals living in them to chronic stressors and also increase their likelihood of acute negative life events such as job loss linked to employment insecurity, episodes of ill-health, and relationship breakdown (Aneshensel, 2015). Lack of access to material resources and discrimination that limits choice of housing for ethnic minority and migrant populations often results in their being concentrated in such socioeconomically disadvantaged areas (Galea et al., 2005). The disadvantage associated with the overlapping, marginalized identities of individuals living in these communities increases their exposure to stressors and results in cumulative adversity, as successive hardships result in increasing inequality in outcomes and access to resources over time (Hatch, 2005). One way this occurs is through a process of “stress proliferation,” where a primary, often acute, stressor leads to further chronic stressors (Aneshensel & Mitchell, 2014; Pearlin et al., 2005).

The stressors common in socioeconomically disadvantaged areas have been shown to increase the risk of suicidal behaviors (Aschan et al., 2013; O’Connor & Kirtley, 2017) both directly and through increasing the risk of mental illness (Aschan et al., 2013). The literature on socioeconomic context and self-harm tends to treat cumulative adversity as “almost inevitably” leading to self-harm (Chandler, 2020, P2). However, studies using the Stress Process Model to understand stress responses more broadly have noted the importance of social context in shaping the relevance of the stressors individuals encounter to valued social identities (McLeod, 2012), with those that undermine individuals’ self-concept particularly likely to result in harmful psychological outcomes (Thoits, 2013).

Community also shapes the resources individuals can call on to cope with stressors: for example, living in a supportive or empowering context can provide a sense of belonging and purpose that enables individuals to develop the personal resource of self-esteem (Thoits, 2013). Or, conversely, living in a hostile environment exposes them to discrimination or abuse that diminishes such belonging and self-esteem (Aneshensel, 2015). Another important example of such resources which increase individuals’ ability to cope is “social support,” for example, the practical and emotional benefits of having others in your life who care about you (Aneshensel & Mitchell, 2014). The Stress Process Model also points to the importance of considering these and other resources at a community level, where they can buffer the impact of stressors, reducing the stress that they cause individuals (Aneshensel, 2015; Pearlin, 1989).

Aneshensel & Mitchell (2014) note that much of the research evidence suggests the relationship between resources and stressors is one of ‘mediation’: that stressors exert their negative effects on mental health *through* the way they reduce individuals’ access to resources. Within suicide and self-harm research, higher rates have been associated with social fragmentation (Congdon, 1996) a concept which refers to a lack of integration, that is social bonds between individuals and groups, within a community (Bearman, 1991; Mueller & Abrutyn, 2016). It is usually measured using population churn and other markers of a less settled community such as the proportion of single person households, which are higher in urban areas of socioeconomic disadvantage (Congdon, 2013; O’Farrell et al., 2015). The underlying assumption is that communities that are less settled and cohesive offer less protective social support and reduce individuals’ sense of belonging (Mueller & Abrutyn, 2016; Wray et al., 2011).

However, studies which have explored socioeconomically disadvantaged areas in the United Kingdom with better than expected health outcomes have suggested resources at a community level could play a positive role. For instance, where such communities provide individuals with a positive identity and sense of belonging, access to informal support, and the ability to take community action against potential harms to health this may buffer the effects of stressors (Cairns-Nagi & Bambra, 2013; Mitchell et al., 2009; Whitehead et al., 2016). The effects of strong social ties within a community cannot be assumed to be universally positive, however. Suicide and self-harm may be increased where high levels of community integration increase the social and psychological costs for individuals who struggle to adhere to community norms (Mueller & Abrutyn, 2016) while strong within group ties can disadvantage those excluded and isolate communities from potentially beneficial external resources (Portes, 1998).

### Setting

The case study area was selected by using clinical data on Emergency Department attendance (Polling et al., 2019a) and hospital admission (Polling et al., 2019b) following self-harm from previous studies to identify small areas with below average age and sex standardized rates of both. Those that were also in the most deprived quintile of the Index of Multiple Deprivation (IMD; Lesser, 2016; a composite measure summarizing multiple dimensions of deprivation which ranks small areas across England), in both 2010 and 2015, were mapped and the largest cluster of contiguous areas selected.

The area selected is in inner South London, approximately 0.4 km^2^, with a population of 6,900 in 2011. The population of the case study area is more diverse than the London average: In 2011, 16% of the area’s population identified as White British and approximately half the population was Black, equally split between African and Caribbean populations. Much of this population has been settled in the area for several generations; the area is one of those known as a center for the Caribbean population who migrated to the United Kingdom in the decades following the second world war. The remainder of the population includes a large number of different ethnicities, including newer migrant communities; overall 50% were born outside of the United Kingdom.

London is diverse in terms of socioeconomic position, with growing inequalities between its richest and poorest residents such that while it has the highest per capita income in the United Kingdom, 28% of households live in poverty, in part driven by the high cost of housing (Raco & Kesten, 2018). This pattern is seen in the case study area. It has been persistently in the most deprived quintile for England from the first creation of the IMD in 2004 up to its most recent iteration in 2019 and two thirds of households are in social rented accommodation. The area has long had a reputation for high levels of crime, drug dealing and use, and gang-related violence. The lower layer super output areas (LSOAs) within the area ranked between the first and 10th centile on the crime domain of the IMD 2015, indicating they still experience very high levels of violent and property crime relative to other areas of England. Alongside this deprivation, recent years have seen increasing private housing developments in the area that are attracting a wealthier and Whiter population.

As is the case throughout the United Kingdom, the National Health Service provides universal coverage of primary and specialist physical and mental health services within the study area, free at the point of access, for all people “ordinarily resident.” Treatment in Emergency Departments is free to everyone regardless of immigration status (Thorlby, 2020). Despite this apparently universal free access, there are persistent inequalities in access to health services in the United Kingdom by race/ethnicity and socioeconomic position (Toleikyte et al., 2018). In particular, Black people in the United Kingdom are known to experience higher rates of compulsory admission and treatment and police involvement in access to mental health care (Wessley, 2018).

## Method

We used a qualitative design, collecting data from interviews and focus groups. We used a “contexualist” approach (Braun & Clarke, 2006) meaning that, while treating the variations in rates of hospital attendance for self-harm which guided the selection of study setting as “real world,” empirically observed findings, we explore the social processes and structures which may account for these findings using an abductive and interpretative approach which understands these potential mechanisms as context dependent (Sayer, 2000). As a theoretical framework, The Stress Process Model was used to inform the creation of the topic guides used for data collection and thematic maps produced during analysis, structuring them around stressors experienced by the community, resources available to cope and to buffer their adverse effects, and the way these elements interact over time to influence mental health and self-harm.

### Participants and Sampling

In-depth semi-structured interviews were conducted with 14 people currently working, paid or as volunteers, in organizations based in or close to the case study area and serving the local population. Two focus groups were conducted with 12 people currently resident in the area. Initially we contacted potential participants within organizations via email and advertised via posters and flyers in community spaces and local groups’ social media. This resulted in recruitment for two interviews, but it became clear that a more personal approach would be required to build trust with potential participants. Recruitment proceeded through face-to-face meetings at local community centers and voluntary sector events, introductions by gatekeepers and snowballing from previous participants. The researcher conducting interviews often met with participants informally prior to arranging an interview. Focus group participants were recruited through the community organizations in whose premises they were held.

Sampling was purposive in terms of the organizations approached, to ensure a range of experience by age, gender, and ethnicity of both interviewees and the people they worked with. We planned a minimum of 10 interviews and two focus groups. The process of recruitment, interviews, and initial analysis was iterative to monitor the identification of new themes and ensure a diverse sample. Recruitment ended after 14 interviews after reaching saturation, with no new themes arising.

### Data Collection

Data collection took place between October 2018 and June 2019. Interviewees chose the location of the interview: 12 were held in their place of work and two their home. Focus groups took place in community centers and participants were given a £10 (approximately US$13) voucher for their time. The amount offered was set to show appreciation for participants’ time, as researchers could not offer a group the same flexibility as it was possible to offer individual interviewees, and the group did not take place as part of their working day. Interviews and focus groups were audio recorded and transcribed verbatim; they ranged in length from 1 to 2 hr.

Both researchers involved in data collection are White women who work for an academic institution strongly associated with mental health services locally. We were mindful of the impact this may have had on participants’ comfort discussing race, gentrification, and critical views of mental health services and the extent to which the researchers felt able to probe these issues. We aimed to mitigate this by taking time to build trust with participants through informal meetings prior to data collection and to reflect on these issues by maintaining a reflexive journal and regular discussions within the research team throughout data collection.

Both interviews and focus groups used a series of open-ended questions with prompts to elicit more information to explore participants’ responses further. The interview and focus group topic guides were the same, covering participants’ views about the area and how it might influence mental health; how people respond when distressed; the meaning, frequency, and causes of self-harm; and how people locally would respond if they had self-harmed. There were no questions about personal experiences of mental ill health or self-harm; participants were asked to think in general about their area and community. The low rates of recorded self-harm in the area were not discussed until the final question in order not to bias participants’ earlier responses relating to self-harm.

### Ethics

Ethical approval for the study was obtained from the King’s College London Psychiatry, Nursing and Midwifery subcommittee (Study Reference: HR-17/18-5744). All participants were given information sheets prior to taking part in the study, which were discussed with them individually at the start of the interview or focus group and provided written consent. An important ethical consideration was ensuring that participants did not experience harm due to discussion of potentially distressing topics. We were explicit about the topics that would be covered in interviews and focus groups both in the written information sheet and one-to-one discussions prior to signing the consent form. The possibility that people might find the subject matter difficult was acknowledged and participants were offered the chance to speak to researchers individually at the end of the session. Materials about accessing help for mental health locally were also offered to all participants. In the focus groups, the importance of keeping the discussion of the group confidential was emphasized in the introduction to the group and agreement to maintain confidentiality of the discussion was included as part of the consent process, as was an acknowledgment that the researchers could not guarantee confidentiality during the focus group.

### Analysis

We conducted a reflexive thematic analysis following the process outlined by Braun and Clarke (2006, 2019). Data were entered into QSR NVivo 12 (QSR International, 2018) and interview and focus group data were coded separately. The full data set was initially coded, and then codes were refined and grouped together through an iterative process of re-reading transcripts and extracts to look for patterns within and across participant responses and produce separate thematic maps for interviews and focus groups. A second rater independently coded four interviews and created an equivalent thematic map. The themes identified by the two separate coders were very similar; together they agreed a combined thematic map. This was compared with the thematic map for focus groups and discussed within the research team who decided to combine the two data sets given the similarity of the themes.

### Sample Description

Interviewees were recruited from youth centers and organizations (5), community centers (3), children’s centers (2), a housing association, faith organization, and school which served the case study area. They largely worked with the area’s established African, Caribbean and poorer White populations, or newer migrant groups. Their ages ranged from 25 to 64 years, 12 were women, 10 were Black (seven Caribbean, three African), and four White (three British, one other European). Ten currently lived in the area, whereas a further two had done so in the past. Focus group participants’ ages ranged from 20 to 64 years, one was male, 10 were Black, of Caribbean (3), African (3), and mixed (4) ethnicity, and two non-British White. Their time living in the area ranged from 1 to over 50 years. Both groups contained a mixture of ethnicities and ages, one was all female and the other majority female. Quotes from interviewees are identified with a P, those from focus group members with an F, but to maintain anonymity quotes are not attributed to specific participants and potentially identifying details have been removed.

## Findings

We identified five interconnected, main themes explaining how the community’s experiences related to rates of self-harm in the area. First, we discuss the *stressors affecting the community* as individuals and collectively. We then consider the resources available to respond to these stressors. At a community level, these arise from *solidarity and a communal understanding of challenges* they face, and a sub-theme of the impact of *population change and fragmentation* on this is discussed. For individuals, a *norm of individual self-reliance* also shapes the meanings stressors have and the resources available to moderate them. We then discuss the *risks of being identifiably mentally ill within the community*, in which we include a subtheme of how *other harmful behaviors* are viewed, and people’s experiences and perceptions of *harm from contact with mental health services*, which together shape people’s behaviors when in distress.

### Stressors Affecting the Community

Participants described local people commonly experiencing multiple stressors related to finances, welfare benefits, employment, housing, and migration status. The cumulative effect of these was often seen as overwhelming, with primary stressors in one domain, like the birth of a child in the example below, resulting in stress proliferation and secondary stressors in other areas of individuals’ lives including greater financial strain and a deterioration in the mother’s health, which also diminish the resources to cope:

INT: Can you give me examples of those kinds of pressures that are piling up?

So, I’m just thinking of one parent in particular, she, and this is the only person I know who self-harmed as well, she speaks English but her husband speaks no English. He works very hard, six days a week. She hadn’t had yet her papers to stay. She has three children, she had a little bit of postnatal depression when she had her second child but got over that quite quickly. Then she had her third child. So they’re still trying to apply for papers, getting in debt trying to finance that . . . [health problems relating to her and her children]. So what started off as low level started to build up because there were so many things. (P)

The sense of having to “get on with it” without much expectation of support intensifies these pressures for new migrant communities under pressure to make their way in a new country with few resources to fall back on:

I would also say that there’s a lot of pressure on kids to make their parents proud and they don’t want to worry them too much and things like self-harm . . . I would say that there is an additional pressure there that perhaps White children don’t face. But I think that’s because your family has moved to a new country, you’re in a country where you’re a minority, the odds are harder. (P)

Beyond circumstances related to individuals’ marginalized social position and specific life events, the experience of living in the study area was seen by many as a chronic stressor. In particular, the pervasiveness of the experience and fear of violence in the area arose in every interview and focus group despite not featuring on the topic guide. Trauma related to this is common in the community and the stress it creates proliferates: Contact with criminality was described as hard for young people to avoid, creating anxiety within families about their young people both being victimized and drawn in as perpetrators. Beyond this high profile “youth violence,” conflict and abuse within intimate relationships and families were also described as common, as was drug and alcohol abuse. The cumulative effect of these area stressors led to the community feeling uncared for by those outside the area and local and national government:

You know your neighbor has been stabbed. You probably know somebody who has been killed. When you’re living in that surroundings, it’s hard to break free from what is becoming normality. And if you’re living it, if you’re seeing a lot of deprivation, and seeing people who are living in poverty, or living on benefits, maybe family members who are taking drugs, it becomes normal and it’s hard not to not do that, if that makes sense . . . I just feel we are creating, for families and people living in this area, being affected by crime becomes the norm. Seeing people who are taking drugs is normal. Living in a place where there’s not a lot of services or community activities that we don’t have to pay for becomes the norm. And it almost is like, does society, does the council, does the government care about me? (P)

For the interviewees speaking about their professional roles, there was an underlying understanding that these stressors resulted in distress for people locally:

I would say it’s a, because it’s such a highly concentrated space, which has had a high concentration of traumatic events, so, and, quite a high level of housing stress. Those are all things that I think mean that a lot of people, I think that a lot of people have experienced a lot of trauma, and feel very stressed and often quite depressed. (P)

All the interviewees described regularly encountering people in mental distress their work, even though none of their organizations had a specific mental health focus.

### Community Solidarity and Communal Understanding of Challenges

Surviving under these circumstances was seen as requiring the ability to protect oneself, family, and community from potential harm and to fight to get what is needed. Participants spoke positively about the way the community had responded to the stressors on it with self-reliance and solidarity, providing them with a feeling of belonging. It had particular importance as a place that provided a sense of ownership for the Black population who did not get to experience this in other contexts:

I like the community spirit. I just like it, it’s alive. (F)

I like that there’s a lot of people who are very similar to me, so there’s a lot of Black people, where as in [city] there isn’t. I love that when I go through [area] I feel like home. (P)

Participants spoke with pride about a history of community action, where local people fought to get the community’s needs met:

[the housing association] are the very same people who watched us slave for 30 years to bring in the £178 million that’s done the estate up. Because they didn’t do anything. We did it. (P)

There’s been a general sense of nobody cares, I think, as in the community will do it for themselves but are other people helping, are other people supporting? Or are they actively undermining any initiatives that would make things better for us, and things like that? (P)

Both these quotes, one from a young, new arrival to the area and another from a much longer term resident emphasize the sense that this action has arisen from necessity as a response to the stressor of external neglect of the area.

#### Population change and fragmentation

The importance of this community solidarity was often most clearly expressed by participants who were describing it as something that was being lost as the area’s population changed. All the participants spoke about the rapidity of this change over the past decade, both due to gentrification and the arrival of new migrant communities. While participants described the vibrancy and diversity of the area in positive terms, these population changes were also seen as undermining the area’s strong community and the sense of ownership it provided to the established population, especially the Black population. Some of this community have been forced to move out of the area by the rising cost of housing, losing highly valued social connections and support as a result. But just as importantly, those that remain feel increasingly fenced in and less visible within the area, parts of which no longer feel like they belong to them:

I’ve seen a lot of changes where my own ethnic group [Black Caribbean] is, like, melting away, you see tons of Europeans and other nations coming in, taking over, you know, stuff like that. (F)

Older participants described how in previous decades the stressors on the community were viewed as collective challenges, but that as the population changed this understanding was shifting toward a more individualized perspective. People spoke about the stress arising from the gap between the ways of living people saw as possible around them and the reality that they were living, and an increasing sense that if you were unable to get on in the pressured environment of the local area this was a personal failing:

INT: And yet there’s something about it that you feel, this change, that’s not for the better, is that right?

I think cohesion has gone. I think, expectations have changed. And we are driven by an agenda that is basically saying, if you’re not able to cut it in London, you get out. (P)

Participants felt that people locally, especially younger people, had been influenced by the general increase in awareness of and openness about mental health issues in recent years. One older participant observed how this formed part of the transformation of social pressures on the community into individual problems:

Sometimes when we use the word mental, it’s words isn’t it? It’s like stress. Back in the seventies, eighties, who was stressed? Did you . . . Well, you weren’t around, but it wasn’t a word that we use is stress . . . “Boy, things hard. Things hard.” When we say, “things hard,” it’s because there wasn’t a leeway to get any further. We still have to forge forward. We still have to put food on the table . . . [] . . . We were trying to get through to the system. The system wouldn’t let us through there. (P)

Another viewed this as not just renaming experiences but driving greater distress:

That’s why I’m saying I think at the moment many, many more Black girls are finding themselves self-harming and thinking about their lack of self-esteem and thinking that they are not good enough. And I don’t feel that was the situation, certainly when I moved to the area . . . . [] . . . Certainly, I did some wellbeing conversations and certainly I did something about stereotypes and labelling, but that was only around stereotypes and labelling of the community, and not individuals. But over the years, I’ve felt that that has really shifted and much more lodged in people’s minds are this sense of lack of self-esteem. (P)

There is a sense that the undermining of community solidarity they observed was also weakening the individual resources members of the community have to cope with stressors.

### Norm of Individual Self-Reliance

Participants described the stressors the community experienced as creating a culture that expected people to respond with self-reliance and placed a high value on individual toughness in the face of adversity:

It’s really, a sort of a, more of a survival, just get through it, survive it, we’re not going to talk about it kind of culture. Especially with the women, they’re really told to just shut up and fucking get on with it, just get on with it. (P)

Participants who themselves lived locally spoke about this toughness with a sense of pride at their ability to survive under such pressure, with some suggesting the experience built a resilience that may protect people’s mental health:

INT: Do you think there’s things about this area that might protect people? That might make peoples’ mental health better?

Yes. There’s a thing around if you can be resilient and all of those things, yes, and actually stay firm and get through it, you find that you’ve got a strong mental capacity. That’s one of the biggest things that come out around here. (P)

Being able to project a tough, self-reliant persona was described as a necessity to protect oneself from being seen as vulnerable and hence a potential target for abuse or exploitation. However, participants noted that this can result in people having a default way of engaging with the world that contributes to the frequency of experiences of conflict and aggression locally, reinforcing the need for others to project toughness:

I do think in general people feel like they have to assert themselves so they won’t be walked all over, so weirdly people shouting and having an argument is a way of protecting themselves from . . . If you feel like . . . If you know that actually hurting me is not worth it, you won’t. (P)

Both interviewees and focus group participants spoke of there being a culture in the area of not speaking about difficulties in general and especially mental health problems, suggesting people would not want to be seen as unable to manage what were common stressors in the community:

But there’s a stigma to having a mental health illness or being depressed or saying I can’t cope. Everyone’s like, oh you drama queen. Everyone’s got to go through this. Come on now. (F)

And I think there’s a feeling of, I guess around the traumatic events, the major things that happen, I’m not sure people have felt very much that that’s a thing that you would get additional support for, because it’s kind of like well everyone’s had that like. So many people on this estate know someone who’s been stabbed, would you actually think that’s a thing you should try and talk to someone about or get some support for, even if it is seriously affecting your health? (P)

The focus group participants illustrated the importance of self-reliance in the way they spoke about their own experiences. While no one spoke about current mental health difficulties, some participants did speak about past ones. When they did so, they framed the causes as social and environmental pressures such as abusive relationships and traumatic experiences. The focus of their accounts was the role of the individual resources and agency in achieving recovery or avoiding more serious illness. Getting better and seeking help in general were framed as being a personal responsibility:

. . . I was very, very depressed. I’m coming from a broken marriage, a child of my own it’s just like it wasn’t working. So, when I came here, I had to sit down literally at my dining table with pen and paper and said right I’ve got to go to the drawing board now. Because looking at my son, I’m looking at who am I going to be in contact with now and I’m saying I need to make this work. I need to snap out of this. So, with the help of my GP and moving to a new area I chose to make it work for me and in no time, I snapped out of it. (F)

This framing allows narratives around mental health that remain in keeping with norms of self-reliance and do not portray the participant as vulnerable.

### Risks of Being Identifiably Mentally Ill Within the Community

Different accounts emerged about how frequent self-harm, when defined as self-poisoning and self-injury, was in the local area. Some interviewees spoke about regularly encountering self-harm in their work with people living locally. These were people whose work involved regular contact with adolescents and young adults, but this perspective was not shared by all the interviewees who worked with young people. Many noted that they had little personal knowledge of self-harm occurring locally. They felt that the organizations that they worked in were not the kind of places where people would talk about self-harm. This reflected their sense of mental health as something more difficult to speak about than the other problems in people’s lives which they regularly dealt with:

No it’s not something that I encounter to be quite honest. I’ve encountered deprivation and lack of money, food and all the rest of it, but when it comes to self-harm, who’s going to tell me? (P)

Despite different views on whether self-harm was occurring, a similar rationale was given to explain either why people would not self-harm or would keep self-harm hidden if they did. Self-harm through injury, overdose or “suicide attempts” were described as something the community would view as clearly linked to “mental illness.” Having self-harmed would be framed as a failure to maintain the self-reliance norm, and people would fear that if this was known about someone they would be seen as weak or vulnerable and feel ashamed for being unable to cope:

I think there’s just a lot of pressure to be seen to be okay and like nothing can touch you, nothing gets through. And so if people think that you’re self-harming, that impacts that perception. (P)

The most extreme example was given by an interviewee who worked mainly with young men. He described how much harder it would be for an individual and their family if they were known to have self-harmed, compared with having been injured by someone else. He gave examples of young people both hiding self-inflicted injury by attributing it to assault and provoking violence from others as an intentional means of self-harm:

There’s a big difference in families where . . . if someone commits suicide, there’s all this thing about he was weak. You know, he has something wrong, how couldn’t we see it? Why didn’t we see it? Wherefore, if he’s murdered, it’s somebody’s killed my child . . . [] . . . You know, he might have been on the streets doing whatever he was doing, some criminal activity. But that’s different compared to you being you know, depressed, weak, lonely, pitiful. They’re the kind of things that people see. (P)

Being known as mentally ill would also decrease a person’s credibility with the community:

Let’s just say that people use mental health as a weapon. When you have an argument the first thing is, you’re mad, you’re crazy, you’ve been sectioned. Who listens to a crazy person? (P)

This discrediting reduces people’s ability to defend themselves from the risks of exploitation and abuse that come with being identified as vulnerable.

#### Other harmful behaviors

The majority of participants described other behaviors that would not fit a research definition of self-harm as more usual responses to distress in this area and had a broader conception of “self-harm” that reflected this. People spoke about substance misuse, provoking or putting oneself at risk of violence, disordered eating, sexual and other risk taking, neglecting or isolating oneself as common in the local area:

I think self-harming, for me, what I’ve actually seen in my time, is basically drink and smoking. Not literally somebody cutting themselves and . . . I haven’t seen that. (P)

So self-harm doesn’t just mean about someone cutting up their body. Some of the self-harm that we have to deal with is kids being reckless to where they will do stuff that will either get them stabbed, shot at, killed or some other way. (P)

There is potential for ambiguity about the intent of this wider group of self-harming behaviors: many were behaviors that participants described as common and so to some extent normalized in the local area. Participants working with young men were particularly likely to describe involvement in violence was a form of self-harm, while those working with women spoke more about disordered eating and isolating oneself. This ambiguity has the effect of making them less immediately identifiable as the result of or associated with mental illness. This may mean they do not breach the self-reliance and toughness norms in the community in ways that make people vulnerable. It also means they do not automatically carry the same risk of bringing people into contact with mental health services.

### Harm From Contact With Mental Health Services

Participants were aware of the mental health services available as a resource in the community and the community workers interviewed often described signposting people to them as part of their role. However, all participants felt that someone who had self-harmed locally would be very unlikely to seek help from mental health services. Some related this to the fear of being known to have a mental health problem and the risks that this could create within the community. However, participants put greater emphasis on the risk of coming to harm from mental health services if help was sought. Participants described people locally experiencing and anticipating that the mental health care offered would not be useful or effective, as in this focus group exchange:

Because you’re going to get mental help, they’re really just guessing to what’s really the problem, so they kind of misdiagnose the problem. Then mental health just spirals out of . . . because you go for help, they didn’t help you and where else do you go? So you still end up with the problem. So they’re guessing as to what’s going on . . . (F)

Or mis-medicate you. (F)

Black participants especially described the mental health systems locally as not understanding the culture of the Black populations they served and so less likely to treat them in ways appropriate to their needs:

They want to put Western methods onto our culture, cultural ways, in order for us to understand about mental health. And I don’t think it completely works. (P)

There was not a blanket rejection of the potential usefulness of mental health services; participants spoke of situations in which diagnosis and treatment with both medication and talking therapy could be useful. However, the idea that misdiagnosis and misuse of medication was common, the fear of coercive treatment including detention in hospital and involuntary medication, and a sense that involvement with mental health services changed people for the worse in ways they did not choose recurred:

People also think about the repercussions of going to the hospital or of, I’ve attempted suicide so now what does that mean? Does that mean I get locked up in a mental institution? Am I going to get sectioned? There is that also because you’ve taken that extreme step it’s seen; there’s that as well. (F)

Mental health services’ position as part of a wider system of services that were experienced as intrusive and coercive meant the risks of help seeking were seen as wide-ranging, especially for a population where many people are dependent on contact with statutory services for their housing and income and where the criminal justice system and child protection services are already experienced as a threatening presence:

I went to school with some really nice middle-class girls. And a few of them took overdoses and their parents then sent them to the south of France to rehab, and stuff like that. Whereas we’d get none of that. We’d get our children taken away from us, would have to go through social services craziness, we’d probably have to go and do some parenting course, we’d have to go to court, we’d probably lose our flats. There is so much more for us to lose if we were to kill ourselves. (P)

The fear of social services involvement after a young person or parent had self-harmed was a recurring theme, especially for young women. Such involvement was experienced as punitive, but also arbitrary and unpredictable. One interviewee spoke about the difficulty this created for women who might want to tell her service about self-harm:

I think people are aware that if I tell a service in this day and age, that things are shared. So, they’ll either try to completely hide it from you, or they’re telling you because they want support. I think there is a general consensus that people know now, if I go to an organization, I can receive support, it’s not just going to stop. (P)

It is an honest admission that services know that the people they work with are weighing the risks and harms when they decide whether to ask for help following self-harm. The implication is that the bar for asking for help is placed higher for those who feel they have the most to lose.

Overall, while participants spoke about support and solidarity from within the community as a potential aid to managing the multiple stressors they experienced, they did not identify the availability of health and social care services locally as a resource that those coping with mental health problems locally would use.

## Discussion

In this study, we have used the Stress Process Model as a framework to help understand why, in the area investigated, high levels of socioeconomic disadvantage did not appear to be associated with high rates of self-harm. This was despite people living within the community being exposed to multiple chronic stressors and often acute, traumatic, life events. Participants described stressors occurring both because of the multiple, overlapping disadvantaged social positions individuals inhabited and because of community wide experiences, especially exposure to and fear of violence. However, the complex relationships between these stressors, the resources available in the community, and experiences of distress helped illuminate why such stress exposure has not translated into greater use of emergency medical services following self-harm. Community resources, shaped by the population’s experience of marginalization, may help buffer the negative impact of stressors on individuals’ self-concept through the way they shape the meanings they attribute to them. Equally importantly, self-harming was not just described as a mental health outcome but also an act that made an individual identifiable as someone with mental health problems, itself another disadvantaged social position. The effect of this would be to increase the individual’s exposure to stressors further, both by increasing their vulnerability to abuse and exploitation in the community and by bringing them into contact with mental health services. People within the community anticipated these services would cause them additional harm both directly and because they are part of a wider system which treats them poorly already and would treat them worse if they were known to have mental health problems.

### Meaning of Stressors

Previous, mainly quantitative, studies examining associations between deprivation and self-harm rates have tended to treat the cumulative disadvantage individuals living in socioeconomically deprived areas experience as inevitably leading to an increased risk of self-harm (Chandler, 2020). This has made it difficult to unpick why some communities do not fit with the overall epidemiological pattern (Byng et al, 2015). Although there is a growing body of qualitative literature within sociology examining self-harm, most qualitative studies of self-harm have been conducted from a psychological standpoint, with an inevitable focus on individual experience (Chandler, 2017) which may exclude discussion of broader social processes that individuals are unaware of or reluctant to acknowledge (Bantjes & Swartz, 2019). In such studies, the link between deprivation and self-harm is understood primarily in terms of the cognitions and emotions of the individuals involved, for example, feelings of entrapment, defeat, or shame (O’Connor & Kirtley, 2017). The findings of this study suggest one way that community context may act to make self-harm (as defined) less likely is through the way it changes the meaning individuals attribute to their deprivation and hence the emotions evoked (McLeod, 2012; Thoits, 2013).

Participants described people living in the area having an understanding of the meaning of the stressors they experienced as a communal struggle. This fosters social resources that help to buffer stressors through mutual support and collective action (Whitehead et al., 2016) and individual resources of self-esteem and mastery through a sense of belonging and an emphasis on self-reliance (Thoits, 2013). Other authors have noted the role that the ideological context individuals exist within may play in shaping their risk of self-harm by highlighting the harmful effects of a neoliberal emphasis on personal responsibility without acknowledgment of the impact of structural factors on the stressors they experience (Bryant & Garnham, 2015; Chandler, 2020). It may be that in the area studied, the collective understanding that their wider political context both neglects and actively harms the whole community reduces the harmful emotional experience of deprivation as personal failure. The majority of the population’s experience as racial and ethnic minorities may play an important role in this: Studies of mental health more generally have found that compared with Whites, people of other races are more likely to attribute stressors such as unemployment and poverty to structural factors beyond their control (Shellae et al., 2019) and such disbelief in meritocracy has been associated with better psychological health (Diemer et al., 2016).

However, it is important not to overstate the ability of these community resources to buffer stressors. Individuals in the area have limited time and material resources to devote to community action and there is a risk of burn out and disillusionment (Whitehead et al., 2016). Social resources that are predicated on connection and solidarity are particularly vulnerable to being diminished by the population churn in the area. Meanwhile, demographic changes in the population may also be transforming the meaning of stressors (McLeod, 2012); for example, where gentrification brings people who have not experienced and do not understand the structural disadvantage of the established population into the area, they may bring with them norms and expectations that feel unachievable, transforming the experience of unemployment or homelessness into an individual-level inability to “keep up.”

### Avoiding Identification as Mentally Ill

The findings of this study suggest that the context of the community studied may also influence both the way distress is expressed, and the help sought. Qualitative studies with people who have received hospital treatment for self-harm have identified that they anticipate that the act of self-harm will result in them being ascribed an identity as someone with a mental illness (MacDonald et al., 2020). Participants in this study echoed this and described how the emphasis on community and individual toughness and self-reliance add to the pressure within the community for individuals to distance themselves from any suggestion of an inability to cope, acting to increase the risks associated with being identifiably mentally unwell. One manifestation of this is individuals in the community responding to distress through behaviors that are not identifiably “self-harm” even if they are harmful. This highlights a limitation of exploring the links between social context and the single health outcome of self-harm. Although the Stress Process Model was originally conceived as a model linking stress to the development of symptoms of mental ill-health, especially depression (Aneshensel & Mitchell, 2014; Pearlin et al., 1981), it has since been expanded to acknowledge the “shotgun spread of consequences of stress across life domains” (Wheaton, 2009). Our findings suggest a strength of this approach may be that it better represents the burden of stress-related harm in a community where other responses to stress are more common (Aneshensel & Mitchell, 2014).

These same experiences underlie the reluctance of those who do respond to distress with self-harm to identify themselves as having done so by seeking help. In the U.K. context of free, universal health care and a case study area with relatively well-resourced services, mental health services might be thought of as additional social resources moderating the impact of stressors. However, participants were consistent in their view that the absence of self-harm presenting to emergency services was not a positive sign of the effectiveness of other services but rather a consequence of the community not feeling these resources are truly available to them. The Black population of the community in particular expected to be stigmatized and discriminated against by services which they did not anticipate would offer them useful help (Memon et al., 2016). Often these views were based on past personal and community experience (Wessley, 2018). These fears meant that contact with mental health services was seen as a stressor rather than a resource in this context.

Studies exploring lower use of mental health services in minority groups have framed toughness norms as a source of stigmatization of mental health problems within communities and a barrier to help seeking and hence a target for intervention (Memon et al., 2016; Shefer et al., 2013). However, this centers the problem within the communities experiencing disadvantage (Tyler & Slater, 2018) and results in a focus on individuals’ behaviors and beliefs without addressing the social structures shaping relationships in which stigma occurs (Pescosolido & Martin, 2015). Our findings emphasize the role of people’s current living circumstances and the wider social environment that shapes them in maintaining the necessity of self-reliance and the desire to distance oneself from mental illness. Participants described the need for toughness and fear of poor treatment by services arising from the experience of living with multiple marginalized identities—including being an ethnic minority, a migrant, a single parent, and living in poverty—as well as community-level experiences of violence and governmental neglect. For individuals living in the area studied, self-reliance and the avoidance of contact with mental health services were often a necessary adaptation that people were using to keep themselves safe.

### Strengths and Limitations

We focused on a specific, small area of South London to explore the effects of individuals’ very local environment on self-harm, rates of which vary between small-areas within London (Polling et al., 2019b). Our findings support this local focus, with participants describing the case study area as having a strong identity and its population living very local lives within it. However, our highly local recruitment strategy, using community spaces and organizations, meant we were unlikely to recruit residents who have a weak identification with the area or who spend most of their daily lives elsewhere. The experiences of newer residents living in private developments who are wealthier and more likely to be White are not represented in our findings.

The pervasive distrust of external authorities and discomfort discussing mental health in the area made recruitment challenging. As discussed in the “Method” section, the researchers collecting data in many ways embody the White, professional population increasingly moving into the area who many participants viewed as a threat to the community. In collecting and analyzing the data, we were mindful of how this might have impacted who was prepared to take part in the research and how open they were prepared to be. The data collected demonstrate that we were at least partially successful in enabling participants to speak about sensitive issues including mental health and race. We were able to recruit a diverse sample with in-depth knowledge of the area; however, ideally the sample would have contained a greater number of men and people aged under 25 years. Sadly, following a violent incident close to the study area, a planned focus group with young men had to be canceled and recruitment, especially of this demographic, became much more difficult. Hence, findings relating to the experiences of young people, especially men, are largely informed by participants who work with or are parents and carers to them.

### Implications

Self-harm is used as an important indicator of the mental health in the population (Public Health England, 2019), with several recent U.K. studies suggesting rates are rising (McManus & Gunnell, 2020; Morgan et al., 2017). When clinical services assess individuals, identified risk of self-harm plays an important role in who is diagnosed and whether they are referred for additional treatment and support (NICE & National Collaborating Centre for Mental Health, 2011). Within the community studied, high levels of stress were linked to a much broader range of harms than those included in a clinical definition of self-harm, including violent victimization, drug and alcohol misuse, and the health consequences of sexual and other risk taking. These outcomes are less likely to be seen as markers of mental health; indeed, they may be more common responses to stress in the community *because* they were less clearly linked to conceptions of mental illness. Given that the processes reducing apparent self-harm in this community were related to the population’s experience of marginalization, considering self-harm in isolation risks allocating resources in a way that compounds existing inequalities and channels more disadvantaged individuals in distress into less therapeutic pathways. Within mental health services, practitioners need to consider that mental distress may increase individuals’ risk of harm in this broader range of ways when establishing thresholds for access to services and treatment. Public mental health policy should consider this wider range of harmful behaviors as potential mental health outcomes warranting interventions targeted at reducing stress and distress in the population. Failing to do so may exacerbate existing poorer access to mental health support for groups including ethnic minorities and those from deprived areas (Wessley, 2018). Similarly, researchers should be mindful of the limitations of using a narrow definition in studies of self-harm and consider the impact on their findings if social disadvantage and fear of harm from mental health services reduce marginalized individuals’ willingness to identify themselves as mentally ill.

## Conclusion

The stressors on the community studied have acted to create norms that prioritize toughness and self-reliance both collectively and within individuals. This has advantages in the way that it has created a communal understanding of the stressors in the community that may buffer some of the emotional impact of deprivation for individuals. However, it also increases the risks from being associated with mental illness by having self-harmed. Alongside community experiences of mental health services as a stressor rather than a resource, this makes individuals in the area less likely to identify themselves as mentally ill by harming themselves in ways recognized clinically as “self-harm” or seeking help when they have done so.

## Notes

1. This combines suicide attempts with acts that are reported as non-suicidal, acknowledging that such acts exist on a spectrum and that degree of intent to die can be ambiguous, both to the person performing the act and clinicians from whom they seek help. This definition explicitly excludes behaviors that do not have the intent of harming oneself, for example, excessive alcohol consumption.
